# Development and application of an environment monitoring system based on IPv6

**DOI:** 10.1038/s41598-022-19929-4

**Published:** 2022-09-21

**Authors:** Xiuhong Li, Xuejie Hao, Lizeyan Yin, Yushuang Ma, Meiying Sun, Rongjin Yang

**Affiliations:** 1 State Key Laboratory of Remote Sensing Science, College of Global Change and Earth System Science, Beijing Normal University, Xinjiekouwei Street, 100875 Beijing, China; 2 Higher Institute of Computer Modeling and Their Applications, Clermont Auvergne University, Clermont-Ferrand, France; 3 State Key Laboratory of Environmental Criteria and Risk Assessment, Chinese Research Academy of Environmental Sciences, No. 8, Da Yang Fang, An Wai, Chao Yang District, 100012 Beijing, China

**Keywords:** Environmental sciences, Environmental social sciences

## Abstract

The widespread use of the Internet of Things (Iot) makes it possible to connect everything but having enough IP addresses is a fundamental requirement of this paradigm. All previous environmental monitoring systems in China are based on IPv4. In combination with the characteristics and requirements of China's atmospheric environment monitoring system, this paper develops a monitoring system based on IPv6 technology. Users can directly access the monitoring equipment through the IPv6 website to view data and configure operations. This paper first introduces the design and implementation of the software and hardware of the system, then introduces the simplification of IPv6 protocol, the transplantation of IPv6 protocol on ARM and the design and implementation of embedded Web server system. The experimental results show that the developed atmospheric environment monitoring system can realize continuous data acquisition based on IPv6 and provide data-driven support for environmental protection management and decision-making.

## Introduction

### Research status of air pollution monitoring systems

Atmospheric environment monitoring has great significance for the prevention and control of air pollution. Therefore, monitoring the atmospheric environment has always been the focus of research at home and abroad. Since the 1970s, developed countries have begun to pay attention to the monitoring of ambient air quality. To date, the United States has established multiple mature and stable air monitoring networks, including State and Local Air Monitoring Stations (SLAMS), National Air Monitoring Sites (NAMS), Special Purpose Monitoring Stations (SPMS) and the Photochemical Assessment Monitoring Site (PAMS)^1,2^, which can be used to obtain data on conventional pollutants in the atmosphere and other pollutants harmful to human health in real time online. In recent years, various sensor networks have been applied to atmospheric environment monitoring in a large number of studies. For example, Kelechi et al.^3^ implemented a low-cost air quality monitoring system using a design from Arduino and Thing Speak. Chen et al.^4^ used low-cost sensor air quality monitoring to study students' exposure to PM_2.5_. Schneider^5^ uses observations and model information from low-cost sensors to map urban air quality in near real time.

The construction of China's air quality monitoring network began in the 1970s, and a series of national air quality monitoring stations were built^6,7^. There are two main domestic detection methods for pollutants in the atmospheric environment, namely, auto-mated monitoring stations and traditional manual detection. The manual detection method requires the staff to directly collect air samples and then detect and analyze the sample gas in the laboratory, which is time-consuming and labor-intensive. Moreover, the result is susceptible to human interference, making the final result unreliable. Automated monitoring sites monitor air quality through complex instruments and equipment and upload air quality data to the central server in real time. However, they possess the dis-advantages of uneven distribution and low density. In addition, the complicated and ex-pensive equipment of national control sites and the high cost of daily maintenance make it impossible to deploy air quality monitoring sites in a large area and with high density, which means that automated monitoring stations cannot obtain air quality data with high temporal and spatial accuracy^8^.

With the maturity of sensor network technology, increasingly abundant communication methods have appeared. In view of the abovementioned problems, the introduction of IoT technology into air quality monitoring has become a research hotspot. Wang et al.^9^ constructed an air quality monitoring system based on partial least squares regression algorithm with the support of national projects. The university student innovation and entrepreneurship project completed by Mu et al.^10^ designed an air quality monitoring system based on multi-sensors. The multiparameter atmospheric environment monitoring system possesses the following advantages: the node collection equipment has a small structure and is easy to install; the network structure is simple; the sensor de-sign adopts a universal interface so that the sensor can be replaced according to different monitoring needs; and it can complete air quality monitoring tasks continuously, in real-time and automatically. Furthermore, by combining data from different data-release platforms, the multiparameter atmospheric environment monitoring system can provide atmospheric environment monitoring data with a large time density and a wide range of space, offering a scientific basis for environmental governance decisions.

### Research status of IPv6 applications in embedded systems

With the exhaustion of IPv4 addresses, there are increasingly more studies on the ap-plications of IPv6. At present, the development of the internet is in a transition stage from IPv4 to IPv6. Since 1996, a series of standards for IPv6 have been released. However, IPv6 has not been widely used for many reasons. Currently, this is mainly reflected in the lack of innovation in applications, slow integration with the current mature product technologies, unsolved transition problems, lack of applications of IPv6 products, small construction scales of IPv6 backbone networks, and poor types of provided services.

In light of the above background, the application of IPv6 to various embedded devices with limited resources has become a current research focus at home and abroad. First, Durvy et al.^11^ researched and proposed the uIP protocol stack for the first phase of IPv6 testing. This protocol stack is specifically designed for embedded IPv6 devices and implements the basic ICMPv6, ND and IPv6 protocols, with only 11.5 KB for all code sizes and less than 2 KB of RAM. However, with a single structure, this protocol stack has a high demand for the cache of embedded devices in specific applications, so there are few practical applications. In specific scenarios, Li et al.^12^ have constructed a quality traceability system of fruit and vegetable agricultural products on the development platform of ipv6 Linux server and HBuilder through the quality traceability identification code of Internet of Things for agricultural products—Hanxin code. Groat et al.^13^ applied IPv6 to the smart grid. The security of information transmission in the smart grid is very important. However, most of the monitoring and control equipment of power grids are embedded systems, which makes them more vulnerable to attacks from intermediate networks. The study involved the design of a mobile device that prevents the power grid from being attacked by the middle network. By combining the characteristics of the mas-sive IPv6 address space with the security of mobile target defense, the MT6D (moving tar-get IPv6 defense) becomes a feasible solution to the security of smart grids. As part of the rapid development of LoRa communication technology in recent years, Weber et al.^14^ pro-posed a communication model called 6LoRaWAN that integrates the LoRa and IPv6 protocols. Combining the low power consumption and long communication distance features of LoRa in the IoT and the advantages of IPv6 in accessing the Internet, 6LoRaWAN provides IoT node devices with a more efficient communication capability, which shows that the practical applications of IPv6 have attracted increasing attention in related research fields.

China's research on IPv6 started early but developed slowly. With the exhaustion of IPv4 addresses, China's demand for IPv6 applications has become increasingly urgent, so China is gradually strengthening its support for IPv6 research.

At present, IPv6 in China is in the early stage of explosion. In the next 10 years, VARIOUS applications of 5G, cloud computing, big data, AI and Internet of Things will be implemented on the basis of IPv6 Now^15^. In 2021, the Cyberspace Administration of China (CAC), the National Development and Reform Commission and the Ministry of Industry and Information Technology issued the Notice on Accelerating the Large-scale Deployment and Application of Internet Protocol Version 6 (IPv6), and all localities began to deploy IPv6 networks intensively. Many universities took the lead in deploying IPV6 networks, and Shi et al.^16^ proposed the end-to-end IPV6 deployment scheme design of college website groups. Zhang^17^, from the IPv6 transformation research group of China Resources Bank, Zhuhai, introduced the IPv6 transformation work of the Internet application system for public service completed by the bank through the upgrade and deployment of IPv4/IPv6 dual-stack link. China's three major operators (China Mobile, China Telecom and China Unicom) have completed THE IPV6 transformation of LTE networks and man networks, and all 13 backbone direct connections have realized IPV6 connectivity^18^. As the infrastructure is ready, the demand for terminal equipment is highlighted, and some progress has been made in the development of terminal equipment and the solution of communication scheme. A large number of devices are currently limited to IPv4, so it is now necessary for terminal devices to support IPv6. In terms of IoT, some studies have applied IPv6 network to IoT design^19^, but there is still a lack of a large number of terminal devices that can support IPv6 network in practical application. In addition, the 6LoWPAN protocol stack, which uses CoAP as the application layer protocol, is also an increasingly used communication scheme in the Internet of Things. The purpose of this scheme is to provide Http request/response services for embedded devices, which simplifies the packet format of Http. However, CoAP differs from Http in that it provides http-like request/response services, not an alternative to Http. CoAP protocol is based on UDP and HTTP is based on TCP to achieve reliable data transmission. CoAP is more suitable for data transmission between local area network devices, and CoAP cannot provide web services supported by Http. This method manages a large number of device nodes by setting up servers with CoAP protocol, and data transmission of devices is com-pleted through CoAP protocol, which is a commonly used communication scheme in the Internet of Things. However, users cannot access the device's functions through the node's IPv6 address^20^.

### Research status of an atmospheric environment monitoring system based on IPv6

With the maturity of IPv6 technology, the application of IPv6 in the field of environ-mental air quality monitoring has gradually become a research hotspot, but the research results do not exhibit strong practical applicability. In research at home and abroad, Ge et al.^21^ adopted ESP32 wireless module as the Ethernet to IPv6 gateway and realized the communication with the cloud platform through IPv6 network to realize the intelligent monitoring system of indoor air quality based on IPv6. Zhu et al.^22^ proposed a way to realize the collection of urban environmental data by integrating IPv6 networks. Rzepecki et al.^23^ designed corresponding experiments to test and analyze the network-ing of IPv6 in sensor networks. Although these tests showed that IPv6 has good ap-plication prospects, they are mostly at the theoretical stage.

This paper uses the results of domestic and foreign research theories to explore the application of IPv6 in atmospheric environment monitoring and to design and implement an atmospheric environment quality monitoring system in the field. This work realizes that each device can have a globally unique followable IP address as a part of the IoT. The IPSec security protocol of ipv6 makes the terminal device more secure. This work solves the problem of discontinuous address space, and facilitates address management by con-catenating device node addresses in a region. With IPv6 there is no NAT. The direct end-to-end communication between devices improves the efficiency of data transmission and makes the node network more convenient and intelligent. It is worth noting that this article describes how to implement ipv6 on a resource-constrained ARM for an HTTP Web server. The data collection end becomes HTTP server, instead of the traditional data collection node can only one-way send to a server and then display data, which not only makes full use of the advantages of ipv6 massive address, but also realizes that each collection node can be individually and real-time access, control and obtain data.

## Structure of the system

**Figure 1:**
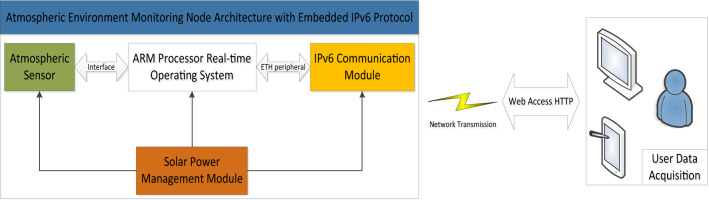
Structure of the system.

When monitoring air quality in the field, the environment is often complex and changeable. It is required that monitoring nodes are deployed with high density and maintained stability to ensure the completion of long-term monitoring tasks. Solar power is mostly used to achieve low power consumption. To meet the requirements of collecting different pollutants, the monitoring node should possess the ability to easily replace components like sensors. This system is mainly composed of atmospheric environment monitoring system nodes with the IPV6 protocol embedded as an IPv6 communication module and data center, as shown in Fig. 1, where the monitoring nodes are responsible for collecting data and uploading the data to the data center.

## Hardware design of the monitoring system nodes

**Figure 2:**
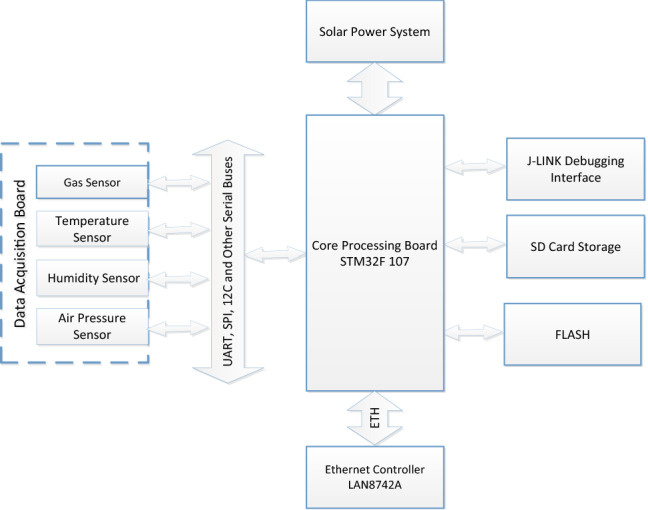
Hardware design framework.

**Figure 3:**
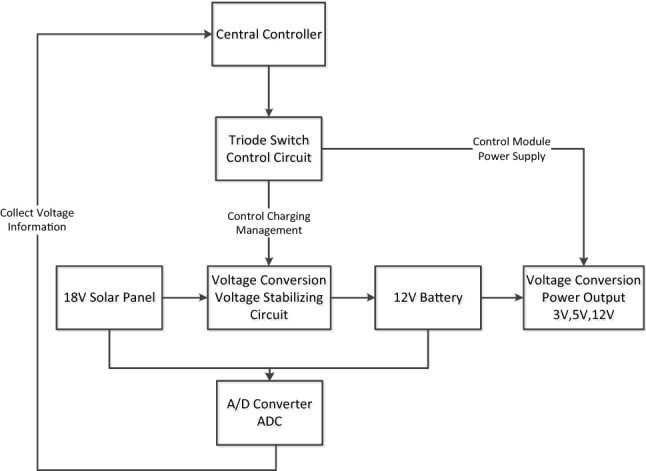
Power management structure of the system.

The overall hardware design framework of the atmospheric environment monitoring system (hereinafter referred to as “the system”) is shown in Fig. 2. Various factors, such as data requirements, energy supplies, and outdoor environments, are fully taken into ac-count in the development of hardware. The modular design is adopted to realize that each module of the monitoring node is connected to the processor. The software controls the coordination of various modules to complete the tasks of data collection, storage and re-mote sending. The monitoring node is composed of modules such as a data acquisition board, a core processing board (the board hardware is shown in Fig. 3), and a smart solar-power management system. The motherboard is the core of the entire platform. The embedded operating system in the MCU on the motherboard performs unified scheduling and coordination of the entire platform to ensure the reliability and adaptability of the system in the actual application environment.

The central controller is the STM32F107 produced by STMicroelectronics, which be-longs to the Cortex™-M3 series 32-bit ARM, with abundant peripherals and ample RAM space. The native STM32 library can provide upper-layer software with interfaces for operating the hardware registers of the STM32 ARM chip, which need to be developed while being used accordingly. In this paper, the BNU embedded operating system embedded in the chip is used to control the whole system, including the processing of the collected data, the storage of the data, the software driver of the communication module, the network communication protocol, the processing of sensor data, the system task scheduling and the controlling of power supply system^24,25^.

The interface of the data acquisition module adopts the serial port method. To connect enough serial port sensors, the system design adopts the method of digital switch polling to obtain sensor data, which effectively reduces the demand for the number of MCU serial ports. The external sensors in this system are electrochemical gas sensors (for SO_2_, CO, O_3_, and NO_2_) and PM_2.5_ and PM_10_ laser-scattering sensors.

SPIFLASH is selected as the data storage module. In practical applications, there may be a temporary interruption in communications. Uploading the data in a centralized manner after storage can reduce not only the frequency of data uploads but also the power consumption of the system.

The power module supplies power to the entire hardware system, combining solar energy and storage batteries to ensure that the equipment can work normally in the field where there is no power supply. Energy savings can be achieved by formulating a corresponding strategy to supply power to the modules when needed and by turning off the power for the modules that are not performing tasks.

The communication module mainly implements the online access to the data publishing webpage through the IPv6 protocol. Users can remotely access the IPv6 address of the device nodes directly through a computer browser, and the node returns web page da-ta, including the data currently being collected in real time, via http, which thus realizes the remote visualization of the collected data. In this way, the need to construct additional servers is also eliminated. Each node is a web server, and the node is responsible for dis-playing the data collected by itself and the operating status of the current equipment.

### Selection of atmospheric sensors

At present, the atmospheric environment monitoring node can be connected to PM_2.5_, PM_10_, SO_2_, NO_2_, CO, and O_3_ sensors through the acquisition board, and additional inter-faces are reserved for access to other sensors. The detection methods for SO_2_, NO_2_, CO, O_3_ and other gases mainly include spectral absorption, electrochemistry and various chemical methods^26–28^. The method of chemical experimentation cannot be applied to online monitoring stations. The infrared detection method is not suitable for large-area monitoring stations due to its high cost of sensors. Therefore, electrochemical sensors are generally used in online monitoring equipment. PM_2.5_ and PM_10_ are particulate pollutants. The traditional gravimetric method and micro-oscillation balance method are commonly used in laboratories, while the laser scattering method can automatically monitor the concentration of particulate matter by using a small and low-cost sensor^29^. A laser scattering sensor is chosen for monitoring in the system. The model and detailed parameters of the sensor are shown in Table 1. The measuring range of the sensor can fully meet the needs of atmospheric environment monitoring, and its shorter response time can re-duce the power consumption of the system.

**Table 1:** Detailed parameters of the atmospheric sensor.

Type	Model	Measuring range	Measuring principle	Resolution	Response time (s)	Accuracy
O_3_	7NE/O_3_-5	0–2000 μg/m^3^	Electrochemistry	2.0 μg/m^3^	< 30	≤ ± 5%
SO_2_	7NE/SO_2_-1000	0–2500 μg/m^3^	Electrochemistry	2.5 μg/m^3^	< 30	≤ ± 5%
NO_2_	7NE/NO_2_-1000	0–2000 μg/m^3^	Electrochemistry	2.0 μg/m^3^	< 30	≤ ± 5%
CO	7NE/CO_2_-1000	0–200 mg/m^3^	Electrochemistry	0.2 mg/m^3^	< 30	≤ ± 5%
PM_2.5_	SDS011	0–2000 μg/m^3^	Laser scattering	0.1 μg/m^3^	< 10	≤ ± 10%
PM_10_	SDS012	0–2000 μg/m^3^	Laser scattering	0.1 μg/m^3^	< 10	≤ ± 10%

### Data store driver

Large capacity storage devices are usually required for embedded system devices to store data. Currently, the generic Erasable Programmable Read Only Memory (EEROM), FLASH (FLASH memory), and Secure Digital Memory (SD) cards can be integrated. The capacity of the first two SD cards is usually smaller than 1 MB. The storage capacity of an SD card is usually larger than 1 GB. The system uses SD card to store sensor data, SD card has the characteristics of removable, easy to read the sensor data directly. In this design, the atmospheric data is single and in order to save processor resources, so only the SD card is used as the large-capacity data storage unit of the node, without complex file system. SD card initialization and single block read and write operation are realized in the system.

**Figure 4:**
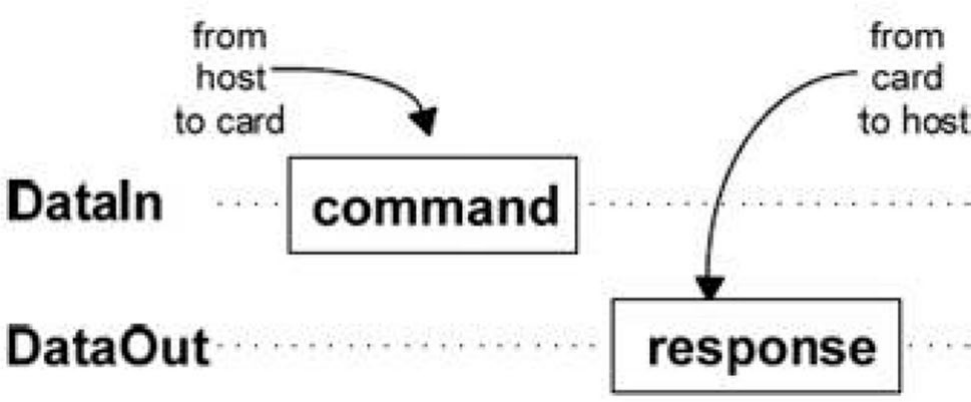
SD card command transfer process.

As shown in Fig. 4, the communication between SD card and ARM adopts the send-reply mechanism, and the data exchange between SD card and ARM is carried out by SDIO bus.

To initialize the SD card, complete the following software configuration:

1. SD default working mode is SD. To simplify read and write operations, it is configured to work in SPI mode. Firstly, STM32F107 initializes the SDIO bus, sets the SPI to work in three-wire system, and sets the operating frequency below 400 kHz.
2. Turn on the SD power, set the chip selection signal CS to high level, and wait for the completion of initialization of SD internal storage, which requires at least 80 SPI clock cycles.
3. The SD card is enabled by lowering the chip selection signal CS. The STM32F107 sends the CMD0 command to the SD card, and the SD card enters the SPI mode.
4. After the CMD1 command is sent and the correct response 0 × 00 is returned, the SD card is initialized successfully and the data transfer begins.

The data transmission of the SD card is mainly completed by command interaction. The data reading process of the SD card is as follows:

1. Pull down the chip selection signal and receive R1 correct response after sending CMD17 command.
2. Send Start Data is received after receiving token 0XFE.
3. CRC integrity check is performed on the received data.
4. Data reception completed, pull up the chip to select the signal, no SD card.

The process of writing data on an SD card is as follows:

1. Pull down the chip selection signal and receive R1 correct response after sending CMD24 command.
2. Data is written after sending the Start Write token 0XFC.
3. Send the data to be written, and then send the two-byte CRC check code for the data block.

Receive data write status, write failure to write again, after the success of the end of the write data, pull up the chip to select signal CS.

### Solar power management system

The system provides a stable power supply for the monitoring nodes by combining solar energy and storage batteries. The optimization of the software procedure can effectively reduce the power consumption of the system to ensure that the monitoring nodes continuously perform monitoring tasks. The smart solar power management system is developed on the basis of an embedded operating system, which can: remotely control the power supply, including turning it on, shutting it off and restarting it (the time point, time period, power mode of “on” or “off”, etc. can all be set); provide 12 V, 5 V, 3.3 V and other DC outputs; protect the system from overcharging and over discharging (the system decides whether to turn off the charging device according to the voltage level of the power supply) and perform other operations. The system can make full use of the solar energy at the installation site to achieve a relatively stable power supply. The performance indicators and characteristics of the smart solar power supply designed for this system are as follows:

40/60 AH rechargeable battery (optional depending on the installation location); A solar panel charging interface that supports 15 W solar panels; Management of charging and discharging of the battery. According to the charging and discharging performance of the battery and the local temperature conditions, the charging voltage and the maximum voltage of the battery can be corrected in real time.

The smart power management system mainly consists of two parts: software and hardware. The hardware structure is shown in Fig. 3. The components are as follows:

1. MCU (Main Control Unit): The MCU controls the entire power module through the in-ternal embedded software, checks the power status of the system through the solar voltage and battery voltage collected by ADC, and controls battery charging of and power supply to other modules of the system through the switch circuit according to different conditions.
2. Triode switch control circuit: The current switch is controlled by the triode, and the central controller controls the triode through changes in the level of the ordinary IO (input/output) port. When the level is high (3.3 V), the switch is on; when the level is low (0 V), the switch is off.
3. ADC (analog-to-digital converter): The ADC converts the analog voltage of solar and battery power into a digital signal quantity and provides it to the central processing unit, provides effective real-time power information for software control, and manages the power module of the system.
4. 18 V solar panel: This panel provides a long-term power supply for the system through renewable solar energy.
5. 12 V battery: A rechargeable battery provides a long-term power supply for the system.

Voltage conversion circuit: The voltage regulator chip converts the input voltage into the required output voltage, and this portion of the input voltage is controlled by the triode switch. The sampling of the power supply voltage is completed by the ADC inside the STM32F107. The ADC converts the power supply voltage into a digital signal so that the software can read the real-time voltage of the solar panel and battery and provide the de-vice's own data status for the autonomous management of the system. According to the voltage data of the battery and solar energy, the device can automatically adjust the sampling frequency of the sensor. When it is judged that the battery power is low, the system reduces its energy consumption by reducing the sampling frequency to ensure that the monitoring time of the device can be maximized without shutting down.

## Software design of the system

**Figure 5:**
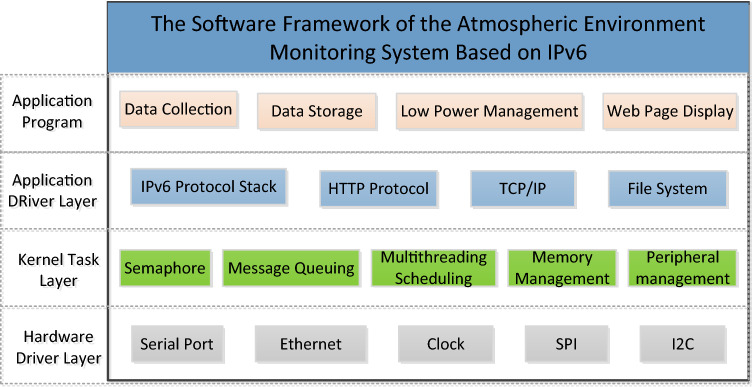
Overall software framework of the system.

The software design framework of the system is shown in Fig. 5. All software runs on the 32-bit ARM processor STM32F107. To monitor the concentration of major air pollutants and particulate matter and display the web page, the software design is divided into four layers from bottom to top: the hardware driver layer, kernel task layer, application driver layer and application program layer.

The hardware driver layer mainly provides ARM peripheral drivers to complete data exchanges between different peripheral hardware components, which is the basic method of external interaction for embedded systems. ARM performs the input and output functions of the chip by connecting peripherals to other devices outside the chip. Each peripheral module usually performs a single function. The peripherals range from simple serial communications to complex 802.11 wireless devices. The software of the hardware driver layer directly reads and writes the hardware registers. The current embedded software de-sign usually uses the standard peripheral drivers provided by the corresponding chip manufacturers directly. The STM32F107 chip selected for the system is the "STM32F1XX_StdPeriph_Driver" driver package provided by the American company STMicroelectronics, which includes the register of read and write drivers for all peripherals of the chip.

As a key layer in the embedded system, the kernel task layer provides basic thread, semaphore and message management functions for the software system. At the same time, it also abstracts the hardware peripherals, which is conducive to calling upper-layer ap-plications. The kernel task layer of this system mainly uses a simple real-time operating system RT-Thread, which contains basic functions to meet different application requirements.

The application driver layer provides functional application drivers such as communication and storage for upper-layer applications and comes with modules such as the IPv6 protocol and file system. These modules directly provide the function interface called by the upper layer so that the applications do not need to rely too much on the lower layer in the actual design and operation of the system and can directly accomplish the tasks that need to be performed by calling the abstract functions.

The application program monitors the node equipment, including the collection and storage of sensor data, management of embedded web servers and minimization of power consumption.

## Key technology

The key part of the system is the remote transmission and display of collected data. This system applies IPv6 technology to the embedded environment monitoring platform of the system and realizes node data visualization through an embedded web server. Taking into account the limited resources, real-time operation and data security of the embedded system, the realization of the IPv6 protocol stack and IPv6-based web server in the embedded system is the key technology of the system design. To enable nodes to connect to the IPv6 network and consume minimal resources, the system uses the LWIP protocol stack, which is a set of open source TCP/IP protocols for embedded systems developed by Adam Dumkels et al.^30^ of the Swiss Academy of Computer Sciences. To date, version 2.1.0 has realized the IPv4/IPv6 dual stack protocol. The lwIP implements the basic communication protocol, but to apply it to a specific system, targeted research is needed, including the porting of the hardware interface layer, realization of upper-layer applications, and joint debugging of the system. This system uses the lwIP protocol stack to access the IPv6 network, and on the basis of this protocol stack, it realizes the HTTP web server for the visualization of the environmental data collection.

### Brief Introduction of the IPv6 Protocol in sensor networks

The IPv6 protocol was proposed to solve the problems of the IPv4 protocol. The IPv6 protocol stack is a well-established standard, which can be understood in related articles^8,31^.

The implementation of IPv6 in sensor networks is very different from those of general network protocol stacks. Table 2 gives the breakdown and detailed content of each layer in the IPv6 sensor network and TCP/IP protocol in the general PC and compares them with the OSI (Open System Interconnect) seven-layer model.

**Table 2:** Comparison of each layer of the IPv6 sensor network, TCP/IP protocol and OSI model.

OSI model	TCP/IP model	IPv6 sensor network	Functions of each layer
Application layer	Application Layer	Simple to Achieve or not Directly	Hypertext Transfer Protocol, File Transfer Protocol, Simple Mail Transfer Protocol
Presentation layer			
Session layer			
Transport layer	Transport Layer	According to the Characteristics of the System, Select the Appropriate Protocol to Implement	Transmission Control Protocol, User Datagram Protocol
Network layer	Network Layer		Network Protocol, Network Control Message Protocol
Data link layer	Network Interface Layer	Network Interface Control Chip Completed	Ethernet, Token Network, FDDI, etc
Physical layer		Drive Circuit	Various Transmission Media

As shown in Table 2, the TCP/IP protocol is simplified to four layers: the application layer, transport layer, network layer, and network interface layer. In the IPv6 sensor network, all layers except the network interface layer are implemented by a pure software protocol stack. The network interface layer in an embedded system is mainly formed by specific network peripherals and PHY control chips to control the transmission medium and data packets^32^.

**Figure 6:**
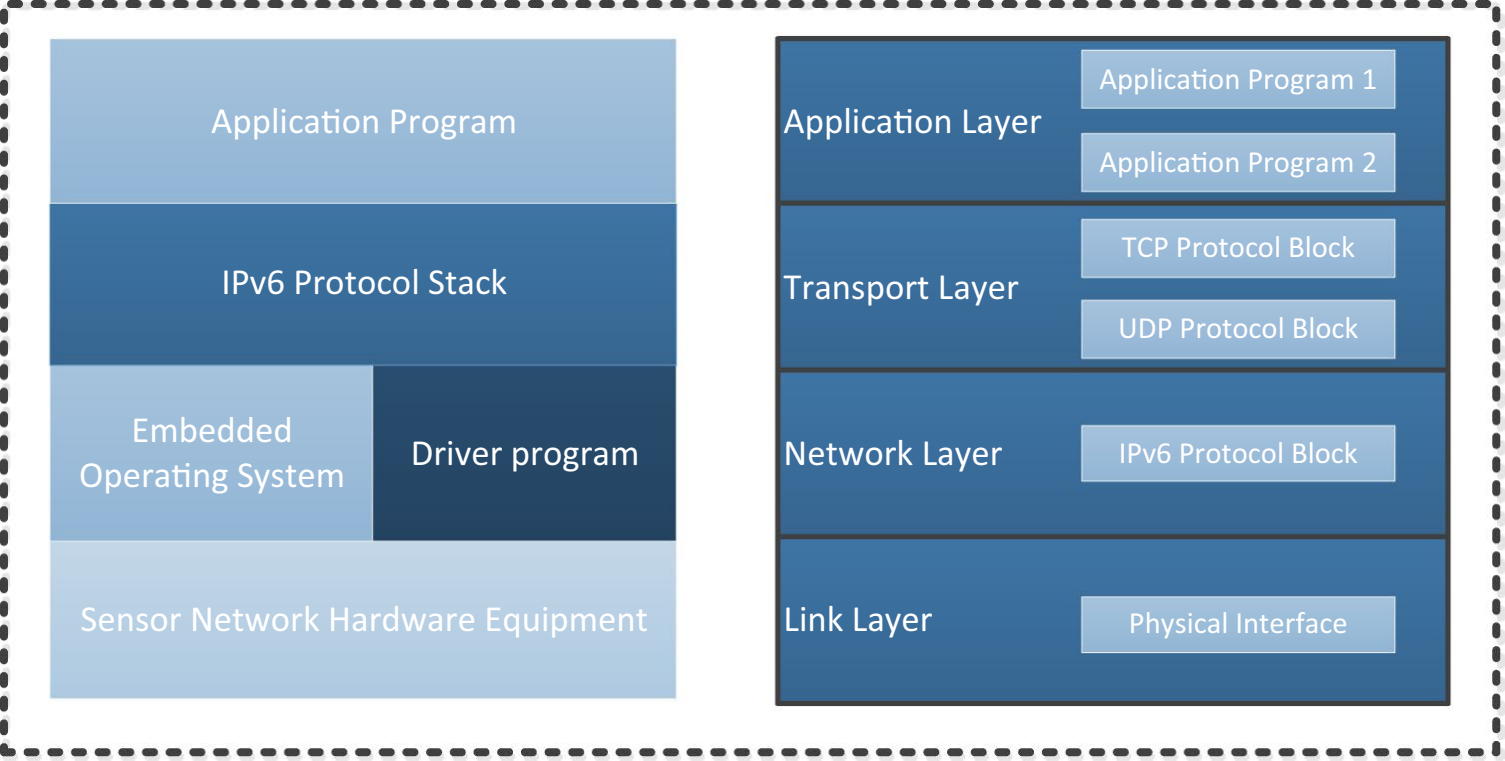
The position and layered model of the IPv6 protocol stack in the system.

IPv6 in the embedded system comprises software and a low-level configuration of hardware peripherals. It has four complete hierarchical functions and contains important components, such as ICMPv6, IPv6 message processing and TCP/IP communication. IPv6 lies between the real-time embedded operating system and the functional program, and its working position in the system is shown in Fig. 6. The embedded real-time operating system provides the basic service functions of thread management, thread synchronization and message transmission for the protocol stack. The upper application and the protocol stack are independent of each other. The application only needs to call the function to satisfy the functional requirement. Therefore, this layered design method is also used in the system to simplify the system design. Under different application requirements, it is only necessary to focus on the design of the upper-layer application, while details of the specific implementation of the protocol stack are not needed. The hardware equipment includes MCUs, ethernet physical layer chip PHYs, and memory. The embedded operating system manages hardware resources through the drivers of the different hardware peripherals, thereby providing services for the protocol stack and other upper-layer applications.

### Simplification of the IPv6 protocol

Simplification of the IPv6 protocol mainly solves the problem of monitoring nodes that access the IPv6 backbone network. Because the hardware storage space and processing speed of the monitoring node are very limited, the original protocol must be streamlined to achieve access at the IP layer. Meanwhile, a compromise is made in terms of carrying capacity and maintenance difficulty.

The simplification of the IPv6 protocol stack in the system mainly involves the connection between layers and the mutual exchange of internal data. The IPv6 protocol stack itself is designed with a four-layer hierarchical structure. In implementation, the more independent the hierarchical modules are, the higher the performance usually is, but the more resource overhead is required. Under normal circumstances, the embedded system has a single application scenario, so strict layering may not be required. When the connections between different layers are implemented, design methods such as function calls can be directly used to save system resources.

The protocol stack processes the message data through the data buffer pBuff. The functions of the data buffer include: (1) providing a management structure for network data packets, which can improve the processing efficiency of packets, and at the same time can improve data processing performance and save memory resources by reducing the number of data memory copies; (2) controlling the throughput of data through the design size of the buffer. In practical application, a buffer of appropriate size is designed in accordance with the data communication requirements of the device to save available memory for other applications in the system. Therefore, the memory management module provided by the protocol stack can better manage the memory resources required by the protocol, reclaim and reallocate the used memory, and ensure that the system will not cause task interruption and failure due to memory resources being run out by network applications. The LwIP protocol stack uses shared memory, message queues and semaphores to connect various layers in order to save resources. Although such a design will lead to less strict and independent layers, it can effectively reduce the resource consumption of embedded devices. The design can fully meet the communication requirements of embedded devices, since they have relatively specific functions, and usually have very clear application scenarios and specific application requirements. The code of LwIP protocol stack is open source. The functional requirements should be fully considered during the porting process, and the buffer size and application modules should be adjusted for different processors and memory resources. The protocol stack adopts modular layering, which enables or disables different modules through global macro configuration to achieve better resource allocation.

### Transplantation of the IPv6 Protocol onto ARM

When the system transplants the protocol stack, the timer is implemented by the timer peripheral inside the STM32F10XX controller to provide time management functions for the protocol stack. The synchronization of this process is realized by the semaphore of the real-time operating system. Therefore, the responsibilities of IPv6 on ARM in the system mainly include providing the functional modules required by the protocol stack, including the semaphore, timer, message mailbox and thread functions; maintaining the receiving and sending interface for data on the network interface layer; initializing the protocol stack; driving the network card of the physical layer; and setting basic parameters for IPv6.

The semaphore is used for task synchronization and resource management. Through the semaphore, events between two independent tasks can be processed synchronously. The message mailbox is an abstract method of message transmission provided by the operating system for the protocol stack, with a similar implementation process to that of the semaphore. However, the memory address pointer of the message is included in the message mailbox delivery process, so the data transfer within the protocol stack can be realized. The mailbox can operate message mailing and message reading. Neither the semaphore nor the message mailbox blocks the process. The task is in a suspended state while waiting for the semaphore or message, and the thread is dormant in this state. When the semaphore or message mailbox is released, it looks for the corresponding thread in the dormant thread queue and then wakes it up. After the thread wakes up, it starts to process the tasks after the semaphore or the message is successfully received. The realization of the semaphore and message mailbox in this system is as follows:

- Semaphore: The err_t sys_sem_new(sys_sem_t *sem, u8_t count) function creates a semaphore. The sem of the semaphore is transmitted by the pointer, and the semaphore function can be provided to the system after it is completely created. There are two ways to call the semaphore in the application thread. One is to limit the waiting time, that is, if the semaphore is not received within the set time, the task is deleted or other operations are performed. The second is infinite waiting, that is, the task is in a suspended state until the signal is received. The u32_t sys_arch_sem_wait (sys_sem_t *sem, u32_t timeout) function is used to wait for the semaphore in the thread. The parameter “timeout” is the waiting time of the semaphore. When the value is set to 0, it means the system is waiting for the semaphore indefinitely.
- Message mailbox: The err_t sys_mbox_new(sys_mbox_t *mbox, int size) function creates a message mailbox. The difference between the message mailbox and semaphore is that the creation of the message mailbox involves opening up a new memory pointer to provide a data transmission channel, while the semaphore does not occupy memory space and provides synchronous processing signals for system tasks through count variables.

The protocol stack also needs to create threads, which mainly include TCP packet processing threads and UDP message processing threads. Therefore, the operating system must provide thread management functions for the protocol stack.

Thread creation: The sys_thread_t sys_thread_new(const char *name, lwip_thread_fn thread, void *arg, int stacksize, int prio) function creates a thread for the lwIP stack. The thread created by the lwIP always exists in the application. For example, the data receiving and data sending processes are divided into two application threads. When the data are not received or are not sent, the two threads are in a suspended state. After receiving the data from the message mailbox, the thread activates the task and starts to send or receive IP packets for processing.

**Figure 7:**
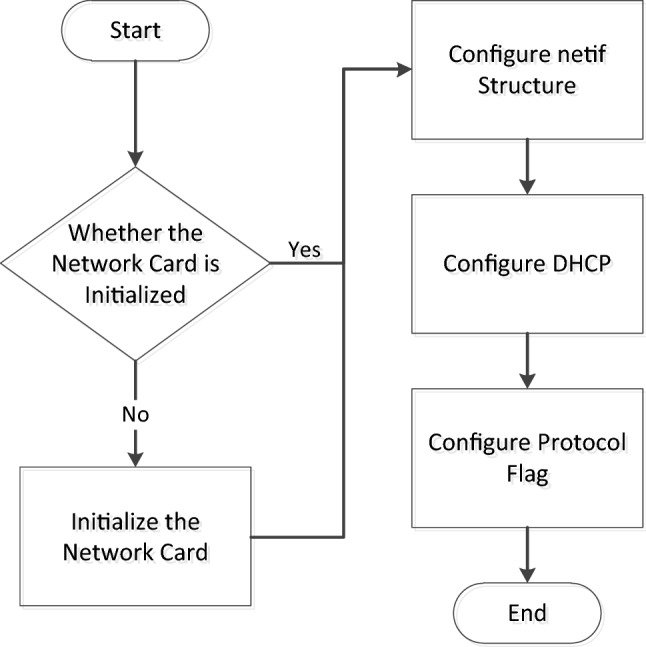
Network driver layer initialization and configuration process.

The initialization of the network interface layer mainly involves the addition of netif to the lwIP stack. The transplantation configuration process is shown in Fig. 7:

Check whether the hardware network card has been initialized successfully. If it is not initialized, then proceed with the network card initialization process.

Configure the network interface, receive and send data, process functions, and write two function pointers into the network structure to provide an interface for the upper layer protocol.

Configure the MLD (multicast listener discovers) to enable IPv6 to discover network devices on the LAN segment.

Configure the DHCP (dynamic host configuration protocol) to automatically configure IPv6 addresses for the device.

Set various protocol flags, call netifapi netif_add, and add the configured netif to the protocol stack to complete the configuration of the network interface layer.

The initialization of the protocol stack mainly creates two thread tasks: data sending and receiving. By creating a corresponding message mailbox for each thread and providing buffer space for the data at the same time, the thread and message mailbox are executed after the creation process is completed. So far, the lwIP has been transplanted on the embedded system. In actual applications, the upper-level user application can directly call related modules for direct use, such as socket connection, HTTP, TFTP and other application modules. This system uses the HTTP module to implement an embedded web server.

### Design and implementation of an Embedded Web Server

#### Introduction to Embedded Web Server Programs

**Figure 8:**
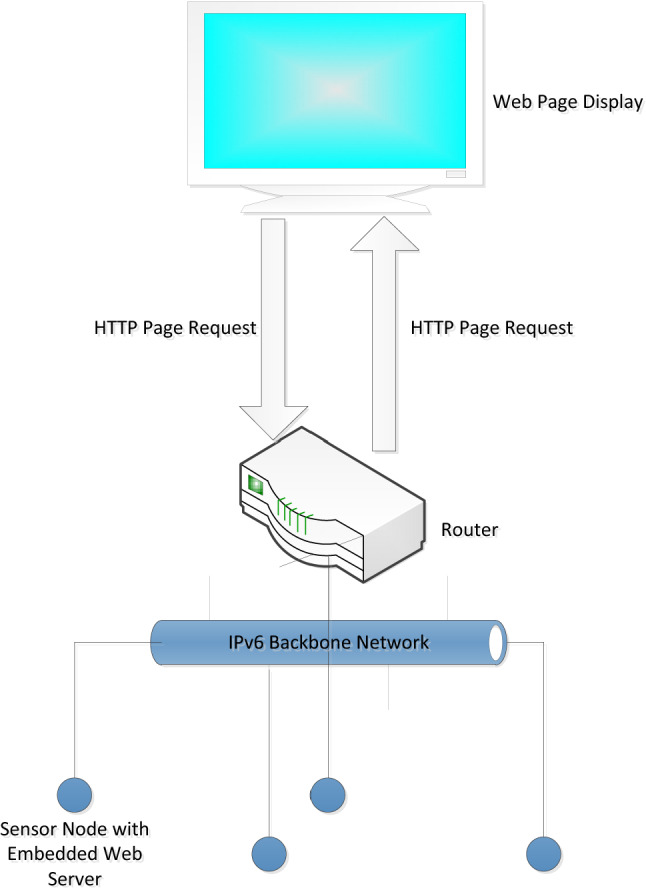
Network topology of the embedded web server.

Unlike the traditional sensor network topology, this system adopts an embedded web server network structure^33,34^. The network connection topology is shown in Fig. 8. All nodes are designed as single web servers and do not require management by a central server. Each sensor node can be directly accessed through a browser to obtain monitoring data and device statuses. This program distributes the traditional background server tasks to each node, and a single node can directly collect, store and display data. On the basis of the rapid development and wide application of IPv6 network technology, each sensor node can obtain a fixed IPv6 address to realize the actual Internet of Things.

#### HTTP webpage design

In embedded systems, there are usually only simple real-time operating systems or direct bare metal programs with limited storage space. Therefore, embedded web design requires tailoring a large number of modules to achieve the desired functions.

**Figure 9:**
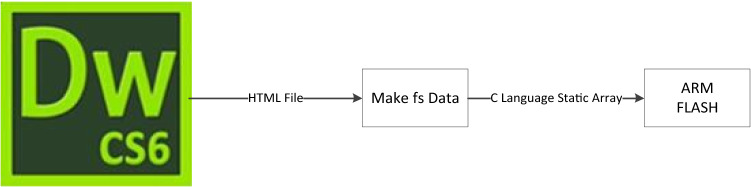
Embedded web page design process.

The webpage of this system is designed with the HTML + CSS + JavaScript architecture. HTML (Hypertext Markup Language) is a markup language rather than a programming language. An HTML text is a webpage. CSS (cascading style sheets) is a programming language for HTML web pages that is used to statically decorate web pages and dynamically format web page elements by cooperating with different scripting languages. Different web page styles can be realized through CSS programming. The JavaScript programming language enables HTML to dynamically request data, change the display text of the web page, and render the display style. Rich, powerful and stable web pages can be displayed through these three languages. The webpage of this system is designed with DreamWeaver software. The specific design process is shown in Fig. 9. First, the web page html file is created on the client computers. The dedicated makefsdata tool is used to convert the HTML file into an ARM-recognizable C-language array file. The array file is stored statically in flash memory or other external memory in the ARM. After receiving the web page connection request, the ARM processor reads the data of the web file. Then, the data is transmitted to the requested webpage through the HTTP protocol, and the webpage automatically parses the HTML file data and then displays the webpage.

**Figure 10:**
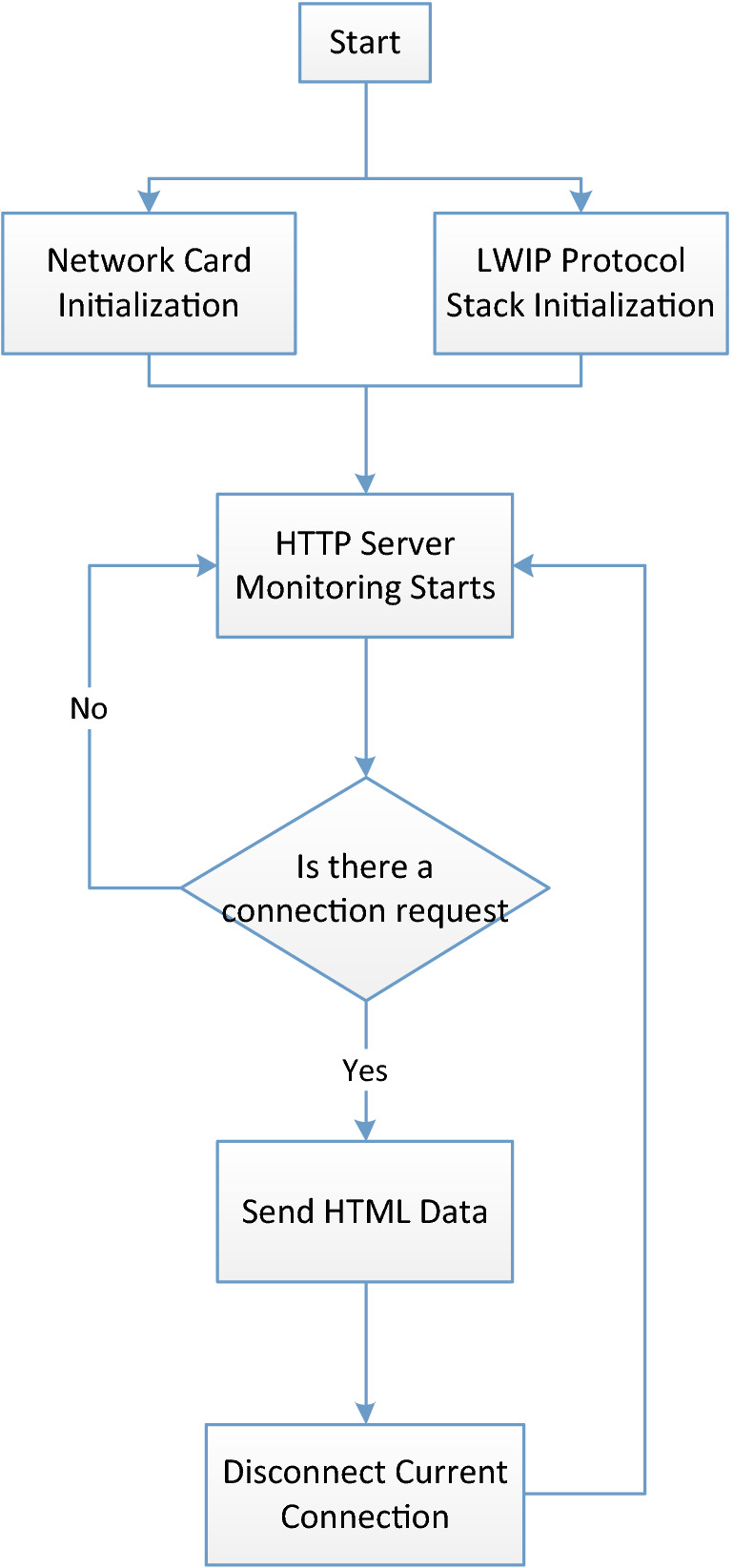
Workflow of the embedded web server.

After the web page is embedded in the monitoring node, the HTTP protocol sends the web page data. Figure 10 shows the actual working process of the web server when the node device is actually used. When the lwIP protocol stack and network card driver are initialized, the software system creates an HTTP monitoring service thread. Users can directly access the IPv6 address of the node through a browser in an IPv6 network environment to obtain the monitoring node's web page. The node uploads static webpage data when it detects an HTTP connection. When the browser receives the HTML format file, it automatically parses the file and displays it. At the same time, the browser parses and executes the JavaScript program in the HTML document and may request the node device to send data again under different functional requirements.

#### Data requests and design display

**Figure 11:**
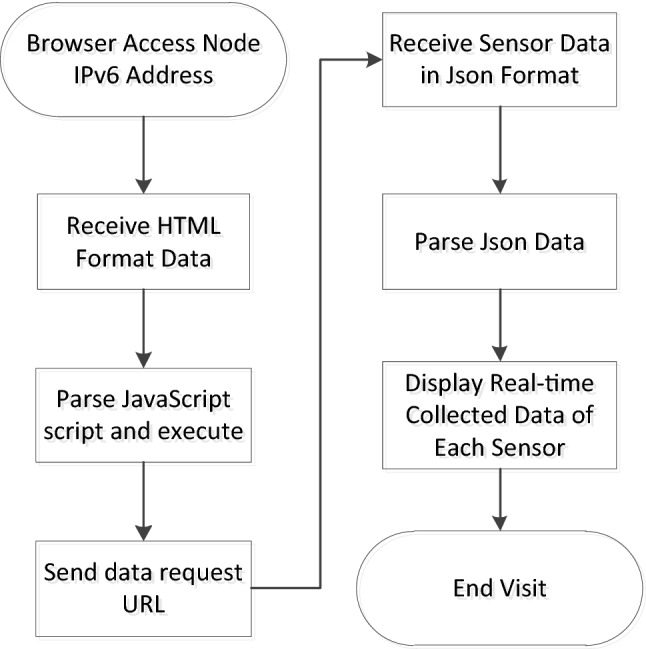
Workflow of the browser connection.

Embedded web pages usually use static arrays to store HTML documents. The data collected each time in this system need to be dynamically displayed on the web page, so JavaScript programs need to be embedded in the HTML document to dynamically request node data. The workflow of the browser connecting to the node once is shown in Fig. 11. The sensor data are sent in JSON (JavaScript Object Notation) format, which is currently a common format used for communication and exchanging data and is independent of programming languages. JSON formatted data are represented by characters, which is conducive to debugging in the development process. At the same time, a standard data exchange format can also improve software compatibility. The format of the JSON package is as follows:

The $.get("./ getair. json", function (data)) command responds to the sensor data in the following format: The data for each field in this example changes based on the actual sensor data.


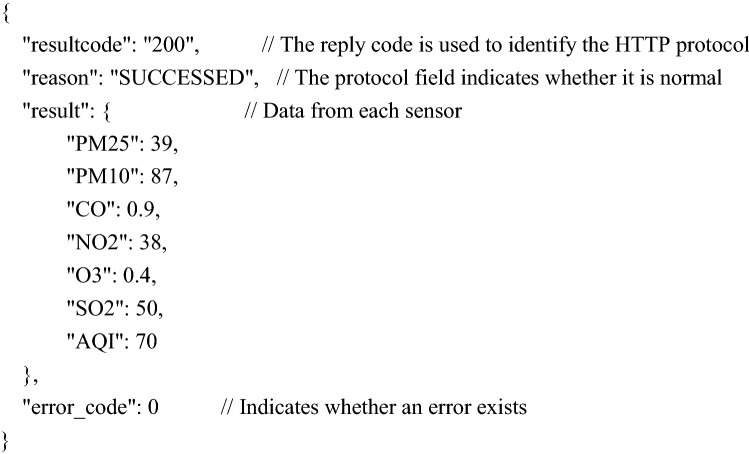


The command $.get("./ getdevice. json", function (data)) requests a reply in the following format (device information, such as ipv6 address).


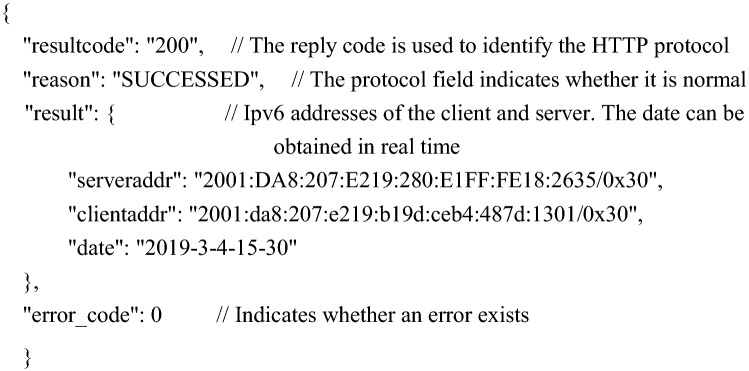


**Figure 12:**
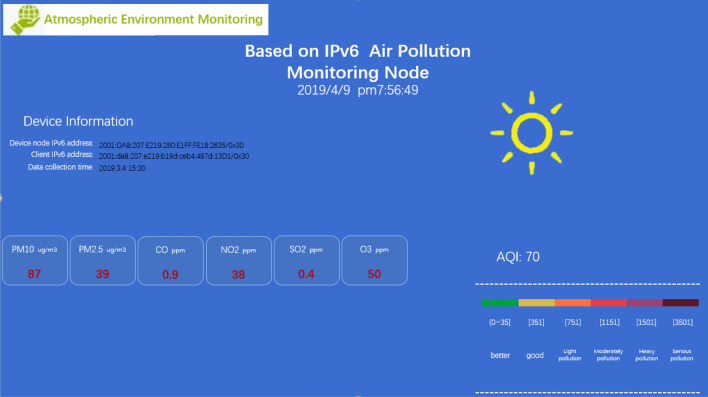
Embedded web interface.

As shown in Fig. 11, at the beginning, users first access the embedded webpage of a node through the node's IPv6 address in the browser. After the node monitors the HTTP connection, it first reads the indexed static web page data directly from the stored data and then sends them. After receiving the HTML formatted file, the browser parses and displays it. To obtain and display the sensor data again, this system adds JavaScript program code to the HTML and sends a fixed URL request to the device. The "$.get("./GetAir.json", function (data))" function requests sensor data from the device. After receiving the request, the device sends sensor data in JSON format to the browser. The browser parses the data and displays it. Then, the browser executes the "$.get("./GetDevice.json", function (data))" function again to request the connection information of the device so that the IPv6 addresses of the device node and client computers can be seen on the web page, which is convenient for users to manage. Finally, the process is executed to obtain a complete display of the web page as shown in Fig. 12, where you can see the nodes accessed through the http://[2001:da8:207:e219:280:e1ff.:fe18:2635]/ URL and the AQI (air quality index) calculated based on sensor data.

## Testing and analysis

### Analysis of the stability test

To verify the operational capability of the system in practical applications, two tests were conducted during this study: testing on the stability and power consumption of data collection by the sensors and testing of the IPv6 network.

The data shown in Table 3 were obtained by monitoring nodes at multiple sites from January to April for 3 consecutive months under unmanned maintenance conditions. It can be seen from the table that the data integrity rate of the equipment was basically higher than 90%, which means that the equipment did not completely shut down during this period of time. The main reasons for the loss of some data are that communications may be incorrectly altered or lost when uploaded or that the device may behave abnormally under the influence of the external environment. However, due to the adoption of a watchdog and timing reset strategy, the device can recover on its own. The results show that the system can meet the requirements of long-term monitoring of the air environment.

**Table 3:** Comparison of the actual data acquisition rates of multiple sites.

Station	TDS	ADS	DAR (%)
Station-1	2712	2712	100
Station-2	2712	2559	94.36
Station-3	2712	2563	94.51
Station-4	2712	2694	99.34
Station-5	2712	2692	99.26
Station-6	2712	2404	88.57
Station-7	2712	2596	95.72

The TDS (theoretical data size) indicates the amount of data that the device should obtain according to the normal acquisition frequency. The ADS (actual data) represents the actual amount data finally obtained. The formula “DIE (data integrity rate) = ADV/TDV” calculates the data integrity of the device.

### Analysis of the system power consumption test

The power supply of the system is a combination of solar and battery power. In the actual test, under the circumstance of low-power operation, the power supply can fully meet the needs of the monitoring system. According to the monitoring data, the device's battery voltage fluctuations basically remained within the range of 12–14.5 V throughout the year, indicating that the device did not experience insufficient power supply conditions.

### Testing of the IPv6 network

The test includes IPv6 network initialization, automatic address acquisition, IPv6 socket communication and an HTTP web service. The test environment is as follows:

- Network environment: Education network (IPv6 campus network).
- Switch: H3C S5100-SI ethernet switch.
- Personal computer: Dell OptiPlex 7040, Window 10 operating system.

**Figure 13:**
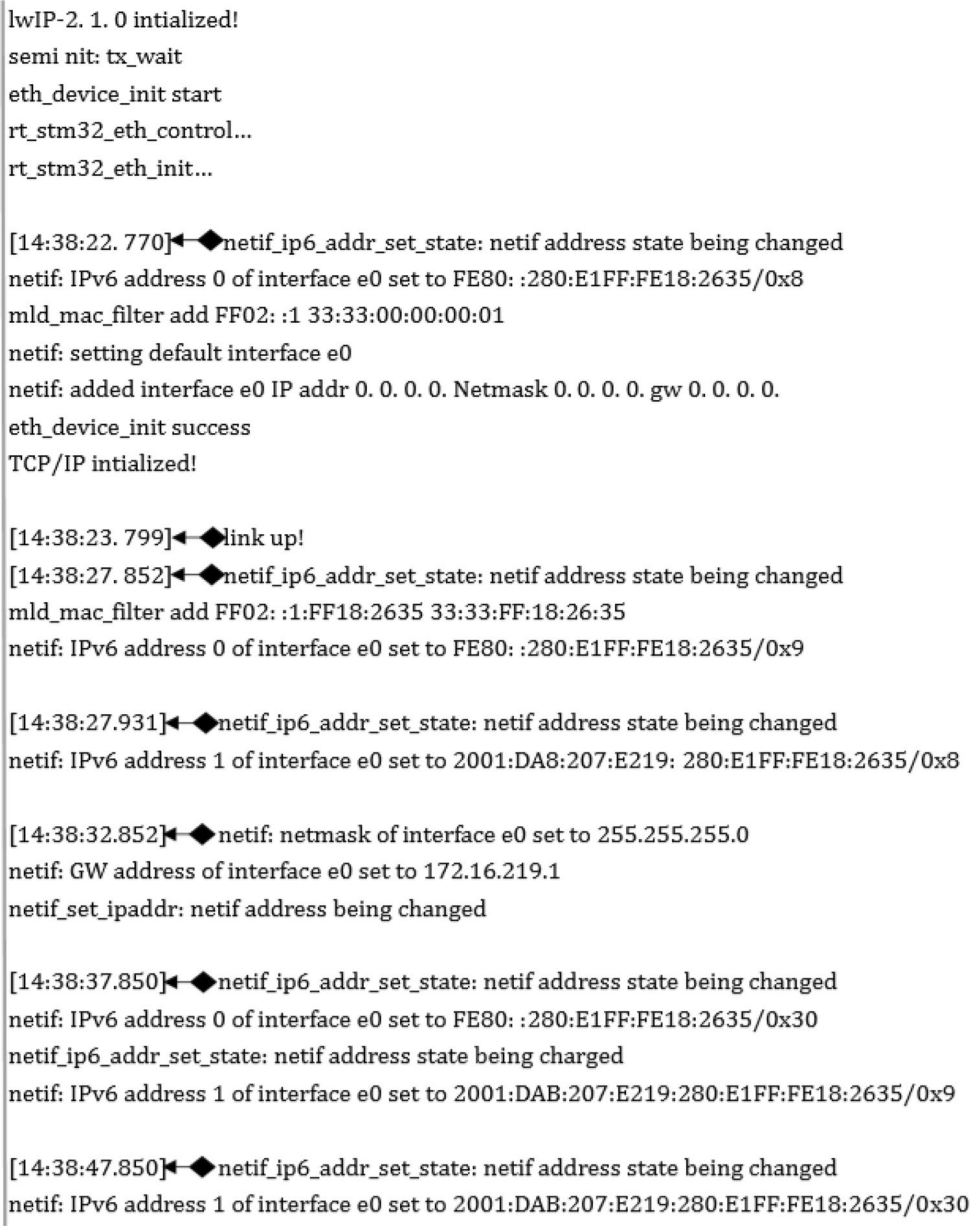
Output Information for system debugging from the serial port.

Compared with IPv4, one feature of IPv6 is automatic state configuration. To test the running status of IPv6 in the system, a function for outputting debugging information is added to the code, the real-time information status is output to the computer through the serial port, and debugging information is output through the computer-side serial port service program SSCOM. The test result is shown in Fig. 13, from which it can be seen that after the lwIP protocol stack is successfully initialized, the system sends out address configuration status information to the router. The switch assigns an IPv6 address, which does not change after the assignment, to the system according to the current LAN IP environment. Therefore, the remote webpage can directly access the embedded server through this address, thereby accessing the webpage that displays the monitoring data. The test results also show that the delay from the initialization of a successful connection by the protocol stack to the reception of the IP address is less than 10 s, indicating that the response speed of the protocol stack in the embedded system can meet the requirements of the application.

**Figure 14:**
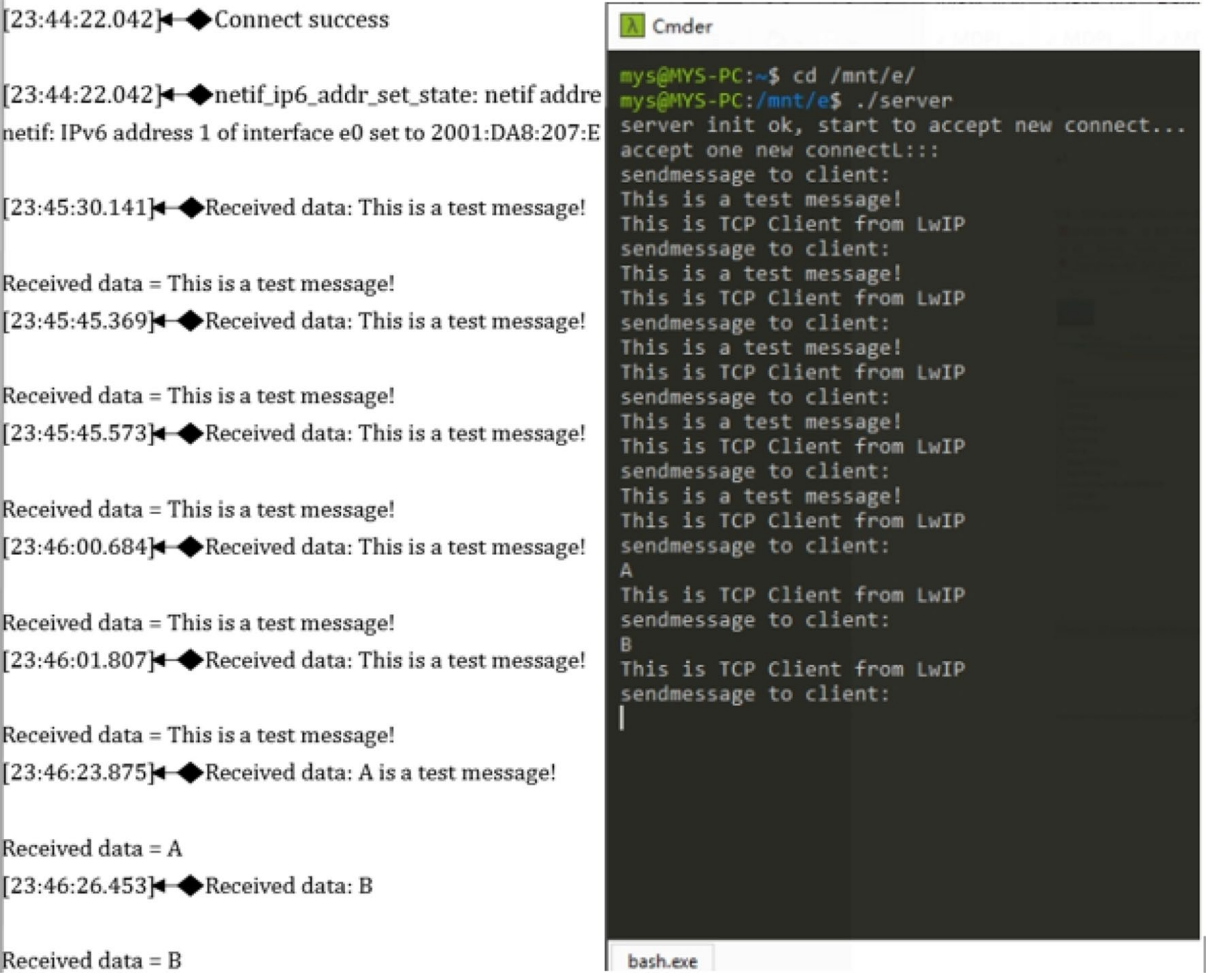
Test information from socket programming communication based on IPv6.

Using a socket is a common way to realize data transmission of two-way communication connections between two applications based on the TCP/IP protocol stack, which is an important component of the protocol stack. Therefore, to test whether the IPv6 protocol stack in this system can support socket applications, a corresponding test module is designed in this system. Furthermore, an IPv6 TCP monitoring server is implemented on the computer side based on Linux programming. In the actual test, the system acts as a TCP client to connect to the monitoring server on the computer. After a successful connection, the server sends test data to the system, and the system client returns feedback to the server after receiving the test data. The test information output is shown in Fig. 14. The test information shows that the system has an IPv6 TCP two-way communication function and that the communication is stable.

**Figure 15:**
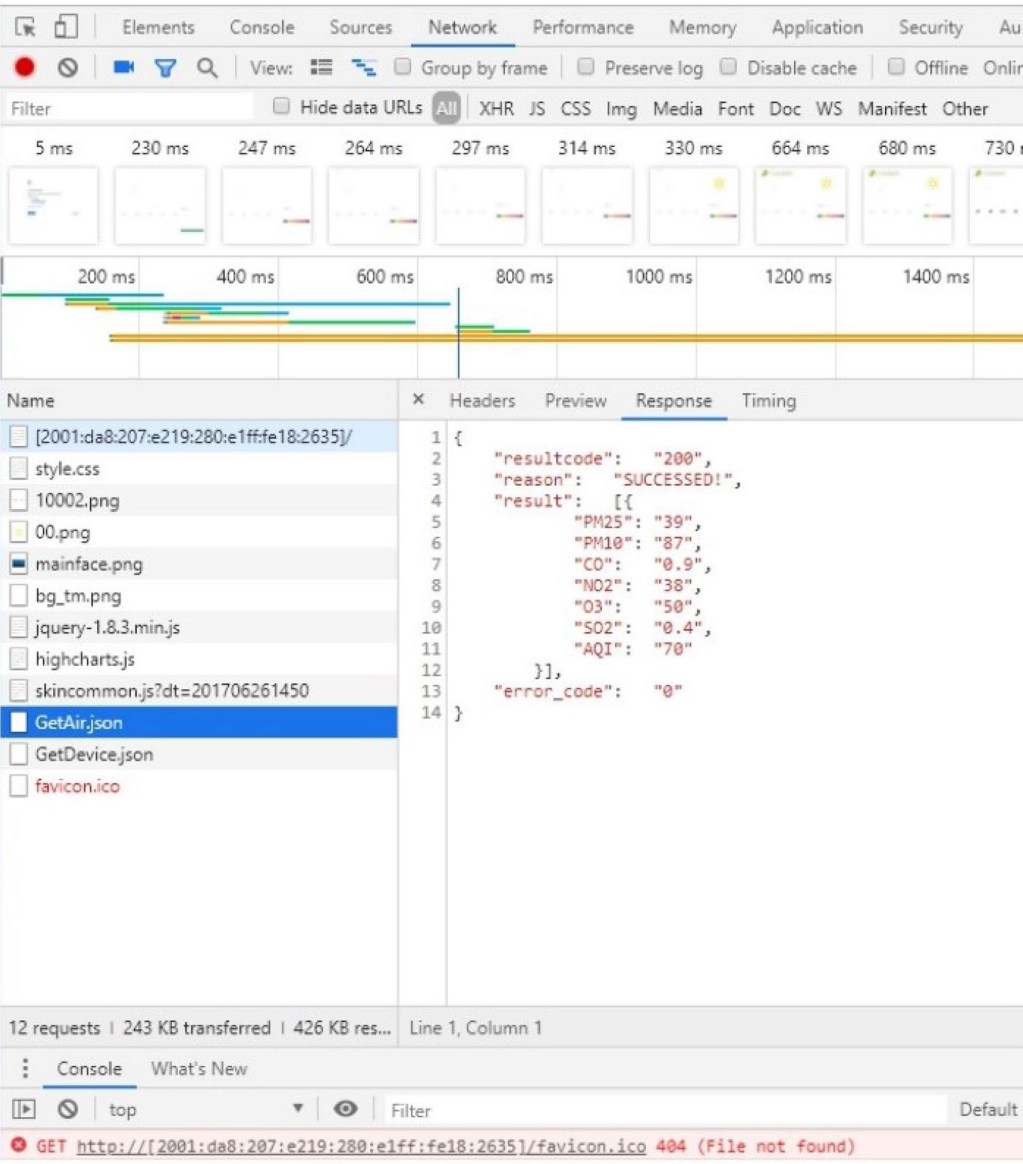
Embedded web page data request to receive test results.

Finally, the embedded webpage of this system is tested. The web page was obtained by directly accessing the IPv6 address of the system through the browser. With the help of the developer tools of the Google Chrome browser, the processes for requesting and receiving data are displayed in detail. The webpage obtained by the test is shown in Fig. 15. It can be seen from the figure that the browser directly accessed the IP address of the system (http://[2001:da8:207:e219:280:e1ff.:fe18:2635]/) to obtain the webpage data and sensor monitoring data in JSON format. In addition, the webpage response time is less than 4 s, which can meet the basic application requirements.

## Conclusion and prospects

Taking the automatic monitoring of atmospheric pollutant gases and particulate matter concentrations as the research background, this paper studies the application of IPv6 technology to the online visualization of atmospheric data in a monitored area and establishes a complete environmental quality monitoring system. According to the system application requirements, the following work has been studied and completed. Based on the 32-bit ARM processor, the system designs the corresponding hardware for the working environment and application of the sensor equipment and completes the miniaturization of the equipment and the remote real-time online automatic environmental monitoring task. The collection and storage of the concentrations of the SO_2_, NO_2_, CO, and O_3_ gases and PM_2.5_ and PM_10_ particulate matter through electrochemical sensors constitute the sensing components of the sensor network. A large-capacity SD card is used to provide storage space for air pollution data and equipment status information. The corresponding storage scheme has been designed to realize the data self-management capability of each node. By transplanting and simplifying the IPv6 protocol stack, the system is provided with network application functions such as sockets and HTTP and can access the IPv6 network of the embedded system. An efficient node management scheme is formed by assigning a unique IPv6 address to a single sensor node. With the help of the large address space and strong security of IPv6, an embedded web page for the atmospheric environment monitoring system is designed. The data requesting function and display design are realized through HTML documents and JavaScript programming. Users can directly access the collected data and device information of sensor nodes through the IPv6 address of the device.

The system adopts a solar power management module to provide an energy supply for the system. A watchdog is also installed to prevent the software system from crashing. According to the actual working environment and functional requirements, corresponding low power consumption strategies are designed. The processor is hibernated under different conditions to realize the low-power management of the system. The verification tests show that the device can support continuous monitoring tasks under IPv6.

There is still much work to be done to realize the Internet of Things, such as the development of a universal interface device, which can easily apply devices under the IPv4 protocol directly to the IPv6 environment.

## Data Availability

The datasets used and/or analysed during the current study available from the corresponding author on reasonable request.
